# Blastic plasmacytoid dendritic cell neoplasm

**DOI:** 10.1002/ccr3.1457

**Published:** 2018-03-04

**Authors:** Manoj Ponadka Rai, Prabhjot Singh Bedi, Samanjit Kaur Kandola, Shilpa Kavuturu, Rashi Singhal

**Affiliations:** ^1^ Michigan State University/Sparrow Hospital 788 Service Road, B301 Clinical Center East Lansing Michigan 48824; ^2^ UPMC East 2775 Mosside Blvd Monroeville Pennsylvania 15146; ^3^ Sparrow Hospital 1215 E Michigan Ave Lansing Michigan 48912

**Keywords:** Allogeneic hematopoietic stem cell transplantation (alloHSCT), blastic plasmacytoid dendritic cell neoplasm, CD4, CD56 and CD123

## Abstract

Blastic plasmacytoid dendritic cell neoplasm is an aggressive neoplasm with a median survival of only a few months despite treatment. An exhaustive immunohistochemical workup is required to differentiate it from myeloid sarcoma and extranodal NK/T cell lymphoma. Treatment is with induction using a regimen utilized for leukemia. Allogeneic hematopoietic stem cell transplantation is recommended for those who achieve remission following induction.

## Case

**Figure 1:**
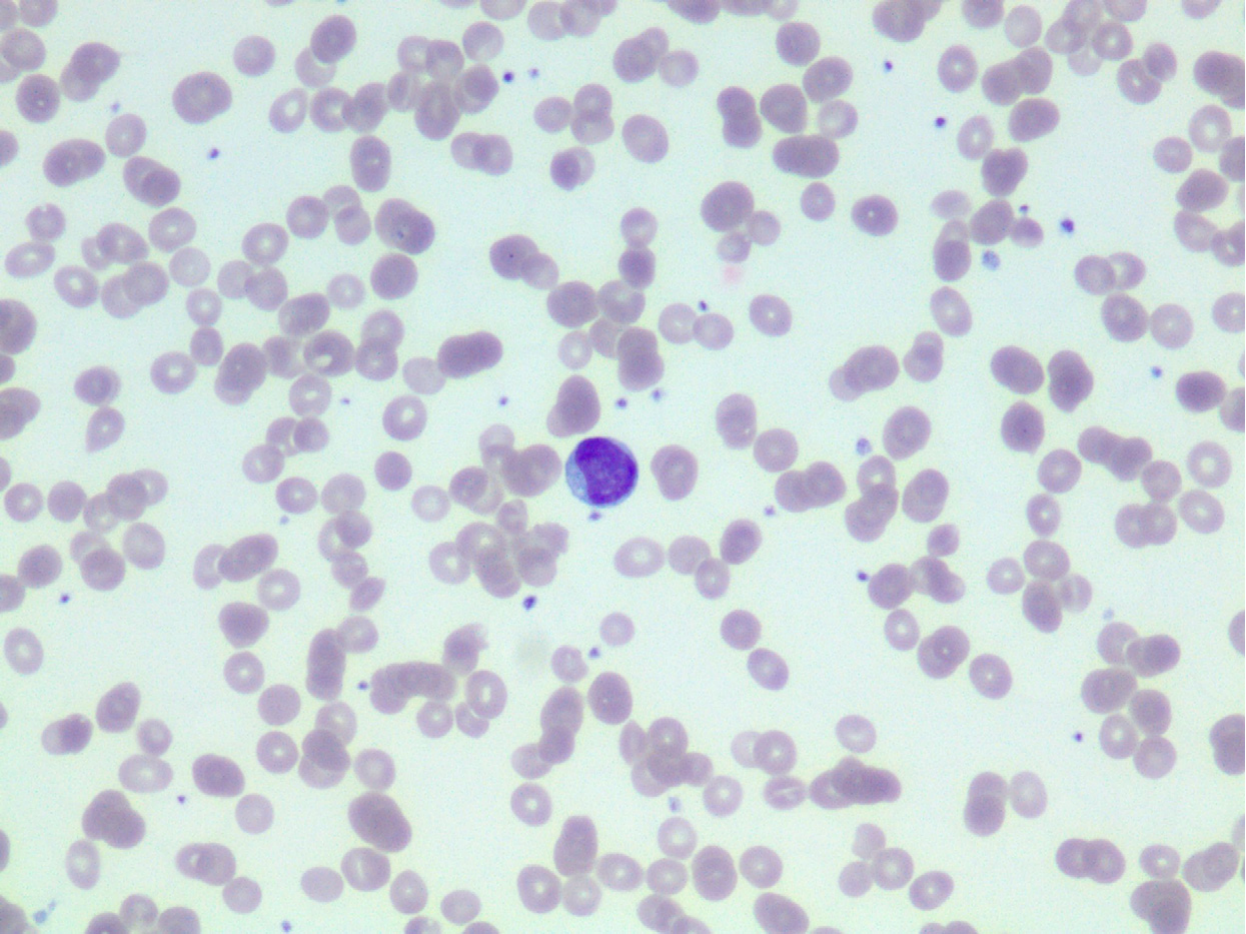
Peripheral blood, smear, Wright–Giemsa stain, 400×: A single blast with ovoid nuclear contours, fine chromatin, conspicuous nucleoli, and a small amount of lightly basophilic cytoplasm with single vacuole and no granules or Auer rods.

**Figure 2:**
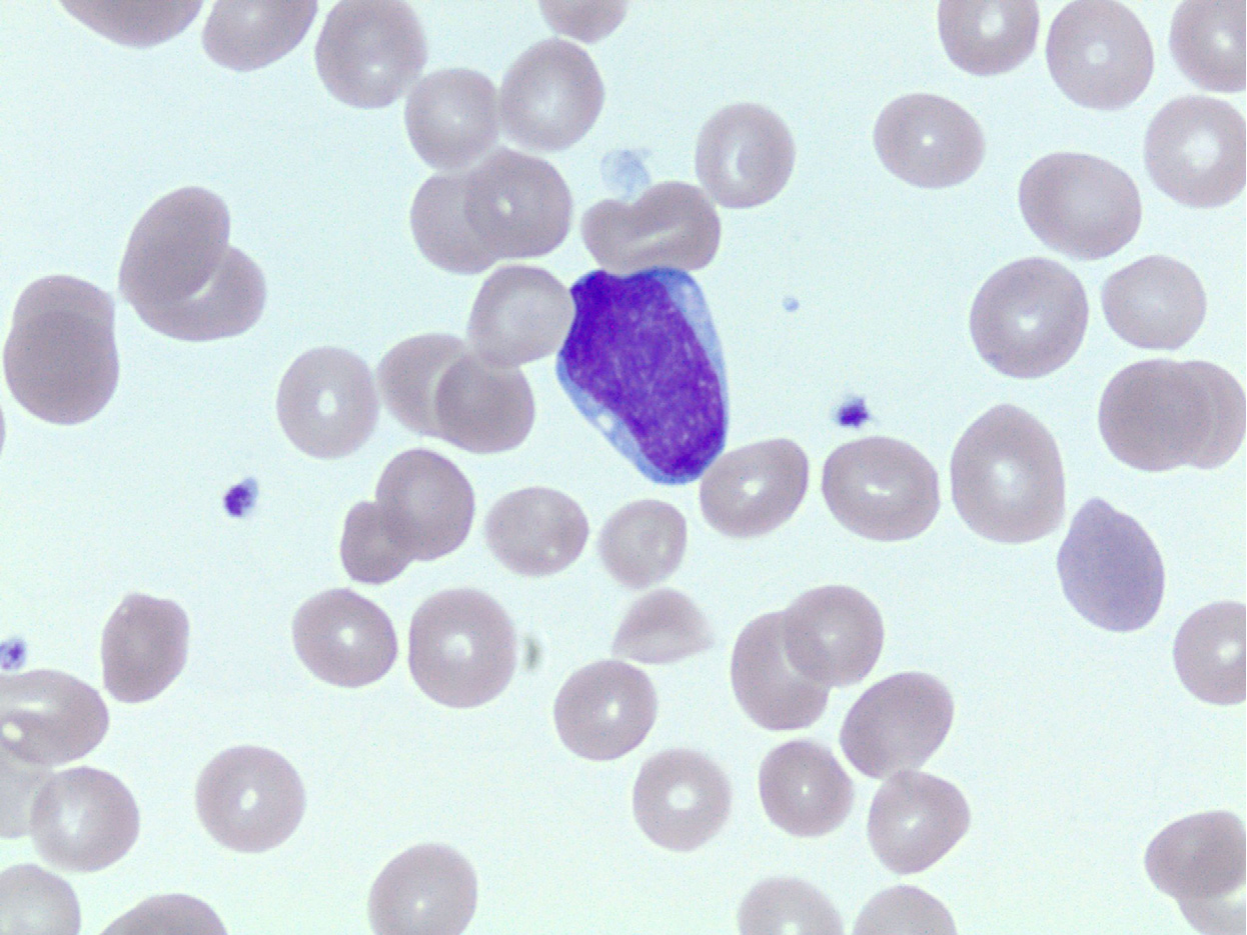
Peripheral blood, smear, Wright–Giemsa stain, 1000×: A single blast with ovoid nuclear contours, fine chromatin, and a small amount of lightly basophilic cytoplasm with a few small vacuoles.

**Figure 3:**
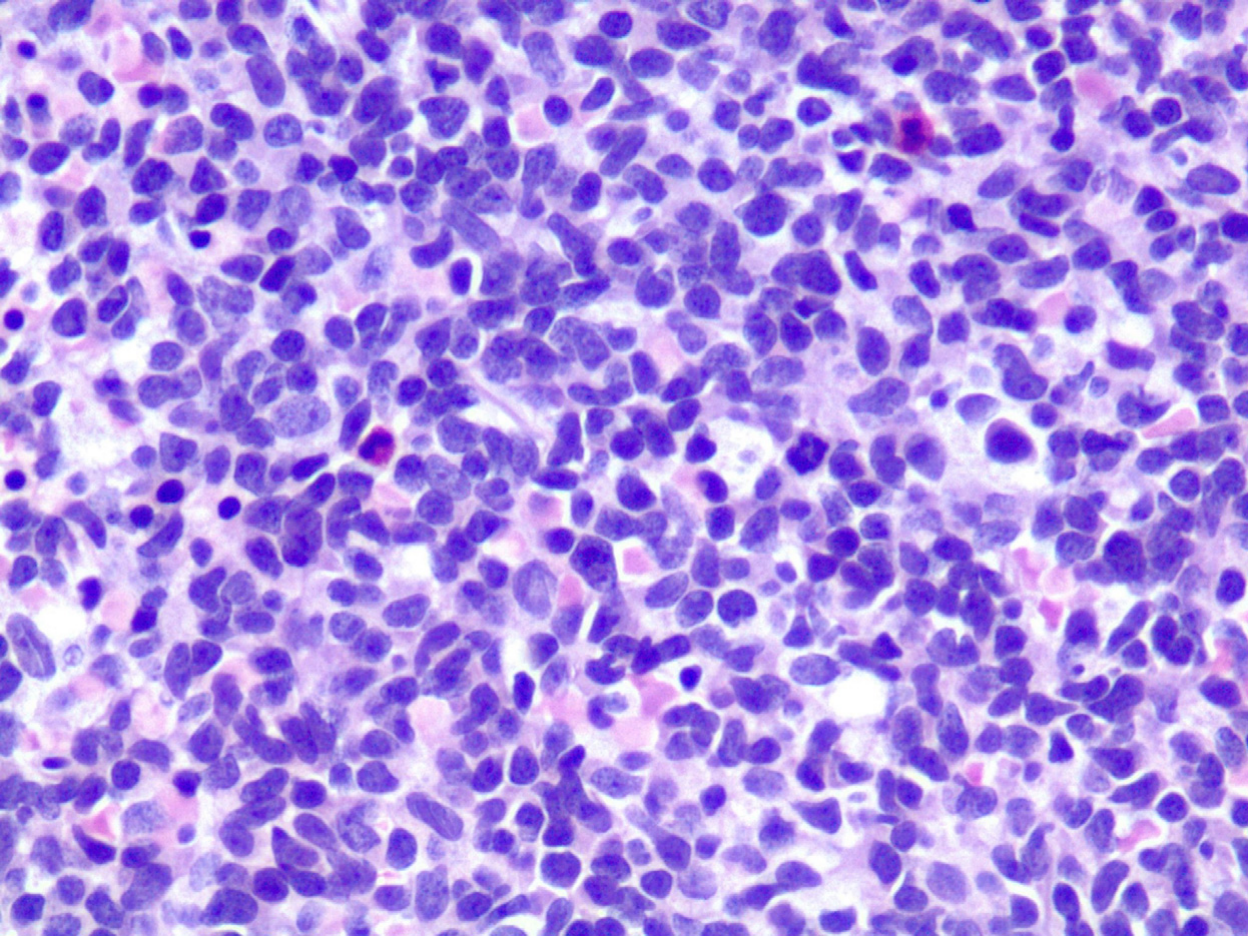
Bone marrow, core biopsy, hematoxylin, and eosin stain, 400×: Diffuse infiltrate of blasts.

**Figure 4:**
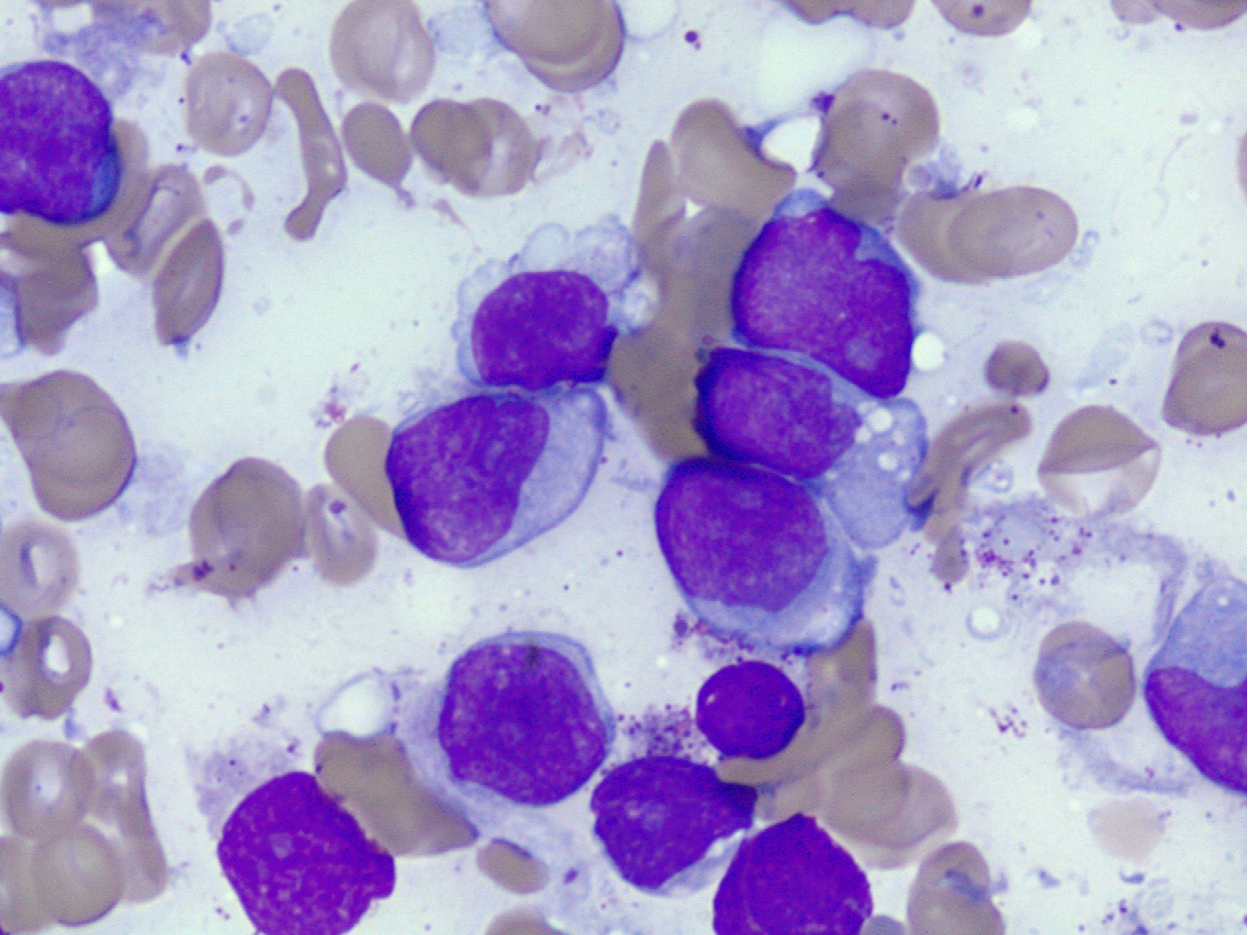
Bone marrow, touch imprint, Wright–Giemsa stain, 1000×: Multiple medium to large‐sized blasts with ovoid and irregular nuclear contours, fine chromatin, occasionally conspicuous nucleoli, and a variably scant to moderate amount of lightly basophilic cytoplasm with occasional vacuoles and no granules or Auer rods.

**Figure 5:**
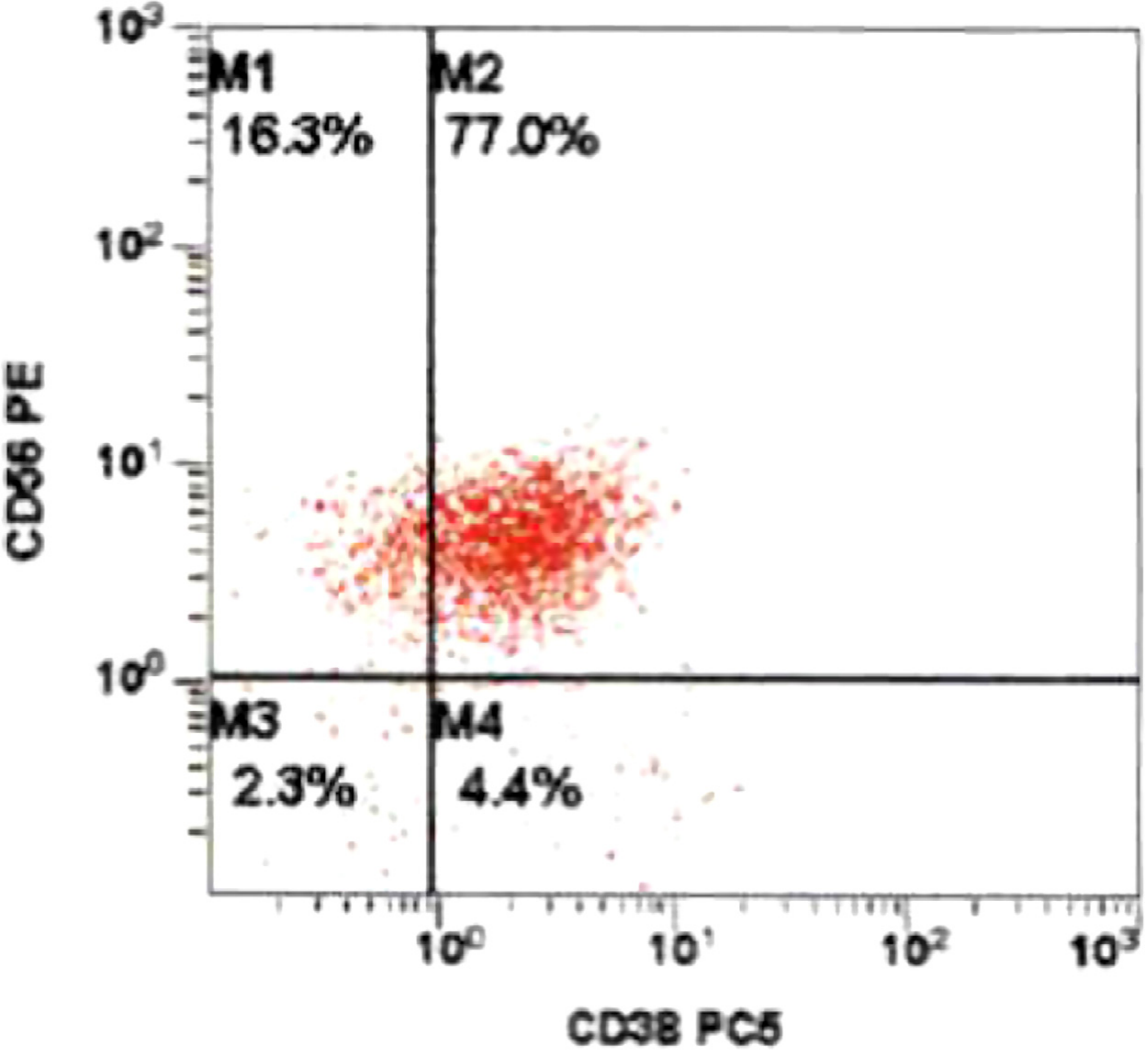
Bone marrow aspirate flow cytometric immunophenotypic histogram demonstrating a cellular population with CD56 and CD38 co‐expression. This population had dim 45 expression, low side scatter, and corresponded morphologically to blasts.

**Figure 6:**
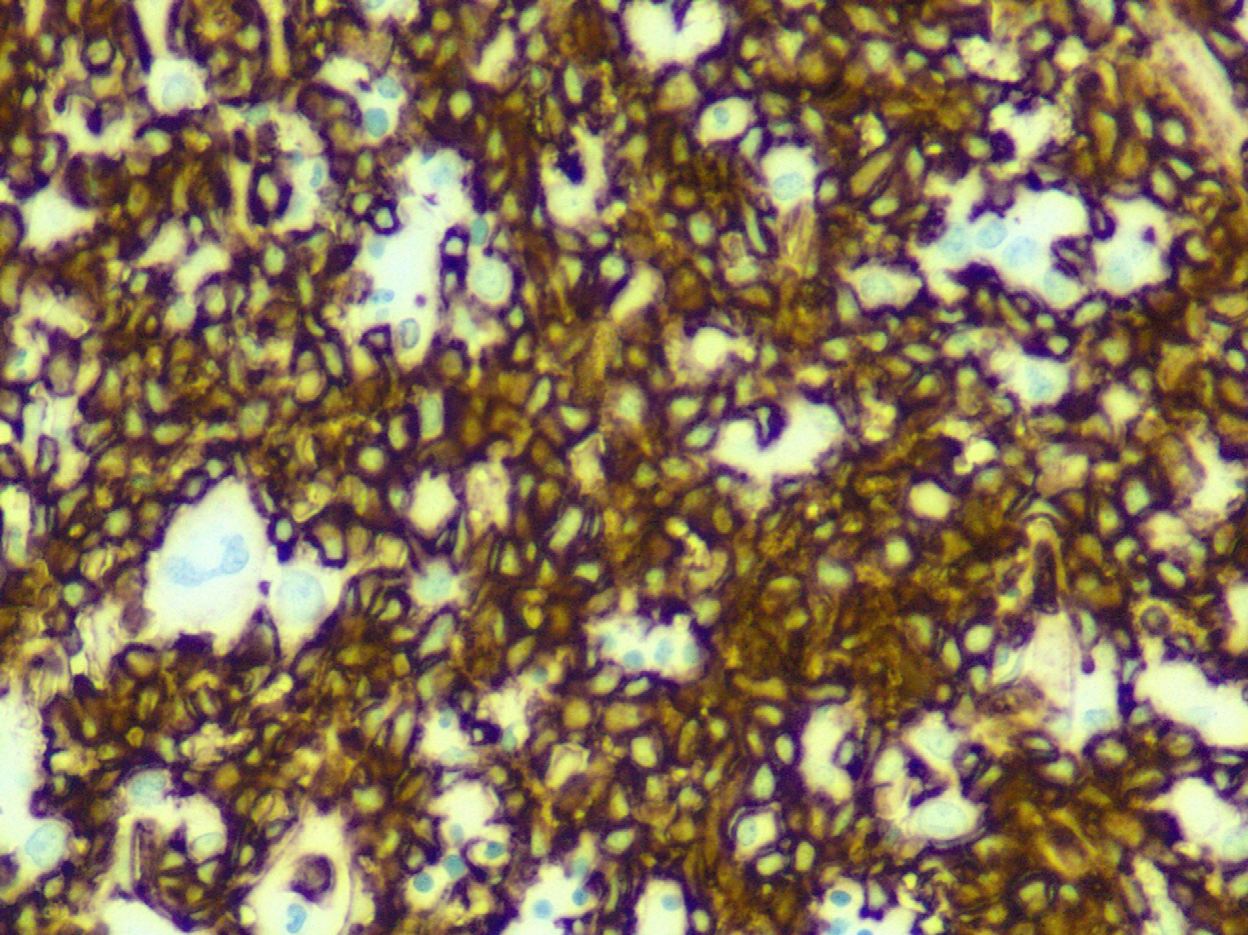
Bone marrow, core biopsy, CD123 immunohistochemical stain, 400×: Diffuse staining among blasts.

A 57‐year‐old male presented to the emergency department (ED) with generalized weakness and severe fatigue. He also experienced constitutional symptoms such as subjective fever, chills, night sweats, diffuse joint pain, and generalized body aches, and was recently treated with amoxicillin at an urgent care clinic for new‐onset dental pain. He completed the course of antibiotics, but his symptoms worsened before he decided to visit the ED. At the time of presentation, he also reported experiencing intermittent “red blotchy” spots on his skin which resolved on their own. Initial vital signs were within normal limits. Physical exam was unremarkable. Labs were remarkable for pancytopenia with neutropenia (white blood cells (WBC) 1.3 × 10^9^/L, absolute neutrophils 0.40 × 10^9^/L, hemoglobin 7.9 g/dL, mean corpuscular volume (MCV) 84 fL, platelets 139 × 10^9^/L. Peripheral blood (Figs 1 and 2) smear showed rare blasts and a bone marrow biopsy was subsequently performed. The bone marrow had approximately 90% blasts (Figs 3 and 4). Bone marrow aspirate flow cytometric immunophenotypic histogram (Fig. 5) demonstrated a cellular population with CD56 and CD38 co‐expression. This population had dim 45 expression, low side scatter, and corresponded morphologically to blasts. Bone marrow aspirate showed immunohistochemical and/or flow cytometric immunophenotypic positivity for CD2, CD4, CD13 (partial), CD33 (partial), CD38, CD43, CD45, CD56 (weak), CD68, CD123 (Fig. 6), HLA‐DR, and TCL1. The blasts were negative for CD1a, surface and cytoplasmic CD3, CD5, CD7, CD8, CD10, CD11c, CD14, CD19, CD20, CD22, CD23, CD30, CD34, FMC7, kappa, lambda, MPO, and TdT. No clonal abnormality was identified by the chromosomal analysis. The morphologic and immunophenotypic features supported the classification of blastic plasmacytoid dendritic cell neoplasm (BPDCN). The patient completed induction chemotherapy in 2 phases, Phase 1 and 2. Phase 1 with prednisone 100 mg/m^2^ PO days 1–8 and 15–21, Vincristine 1.5 mg/m^2^ (max 2 mg) intravenously (IV) on days 1, 8, 15, and 22, cytarabine 3 g/m^2^ IV over 3 h bid for four doses on days 1 and 2, methotrexate 1 g/m^2^ continuous IV infusion (CIV) over 36 h on day 15, leucovorin 30 mg PO or IV starting 42 h after starting methotrexate every 6 h for six doses, L‐Asparaginase 10,000 units/m^2^/dose IV on days 3, 4, 16, and 17. Phase 2 with Dexamethasone 40 mg/day PO on days 1–4, 9 to 12, ifosfamide 500 mg/m^2^/day IV on days 1–4, vincristine 0.4 mg/m^2^ (max 2 mg) CIV on days 1–4, adriamycin 12 mg/m^2^ CIV on days 1–4, intrathecal therapy with methotrexate 12 mg on day 1 or 2, cytosine arabinoside 30 mg on day 1 or 2 and hydrocortisone 15 mg on day 1 or 2. He subsequently developed neutropenia and sepsis which progressed to septic shock, and he unfortunately expired. BPDCN is an aggressive hematological malignancy derived from the precursors of plasmacytoid dendritic cells. CD4, CD56, and CD123 expression are characteristic of BPDCN 1. CD43, CD45RA, and CD68 are positive in 50% cases. HLA‐DR, CD7, TdT are positive in about one‐third of cases. BDCA2/CD303, TCL1, CLA/CD162, MxA, and CD33 can be positive. Lack of CD56 does not rule out BPDCN if CD4, CD123, and TCL1 are positive. In rare cases CD2, CD36, and CD38 are positive. Myeloid sarcoma and extranodal NK/T cell lymphoma is known to express CD56 ± CD4. However, CD7 and CD33 is commonly expressed and CD3, CD5, CD19, CD20, CD79a, lysozyme, and myeloperoxidase are negative in myeloid sarcoma and extranodal NK/T cell lymphoma 2, 3. Hence, an exhaustive immunohistochemical workup is required to make a definitive diagnosis of BPCDN. The median survival is only a few months as the tumor exhibits a progressive course despite an initial response to chemotherapy 4. Patients receive induction chemotherapy with a regimen similar to that used for acute myeloid leukemia (AML) or acute lymphoid leukemia (ALL). For adults who achieve a complete remission, allogeneic hematopoietic stem cell transplantation (alloHSCT) is recommended 4.

## Conflict of Interest

None declared.

## Authorship

MPR: wrote the case description. PB, SKK and SK: contributed to the description on AITL and the key clinical message. EBM: provided the description of the pathology images.
